# The patient experience of skill mix changes in primary care: an in-depth study of patient ‘work’ when accessing primary care

**DOI:** 10.1093/pubmed/fdad203

**Published:** 2023-12-21

**Authors:** Elizabeth Dalgarno, Imelda McDermott, Mhorag Goff, Sharon Spooner, Anne McBride, Damian Hodgson, Ailsa Donnelly, Judith Hogg, Kath Checkland

**Affiliations:** Department of Public Health, School of Health Sciences, Faculty of Biology, Medicine and Health, The University of Manchester, Manchester M13 9PT, UK; Health Organisation, Policy and Economics (HOPE), Centre for Primary Care Research, School of Health Sciences, Faculty of Biology, Medicine and Health, The University of Manchester, Manchester M13 9PL, UK; Health Organisation, Policy and Economics (HOPE), Centre for Primary Care Research, School of Health Sciences, Faculty of Biology, Medicine and Health, The University of Manchester, Manchester M13 9PL, UK; Health Organisation, Policy and Economics (HOPE), Centre for Primary Care Research, School of Health Sciences, Faculty of Biology, Medicine and Health, The University of Manchester, Manchester M13 9PL, UK; Institute of Health Policy and Management, Alliance Manchester Business School, University of Manchester, Manchester M13 9PT, UK; The University of Sheffield, Management School, Sheffield, South Yorkshire, S10 2JA, UK; The patient and public involvement and engagement group at The Centre for Primary Care and Health Services Research (Primer), The University of Manchester, Manchester, M13 9PL, UK; The patient and public involvement and engagement group at The Centre for Primary Care and Health Services Research (Primer), The University of Manchester, Manchester, M13 9PL, UK; Health Organisation, Policy and Economics (HOPE), Centre for Primary Care Research, School of Health Sciences, Faculty of Biology, Medicine and Health, The University of Manchester, Manchester M13 9PL, UK

**Keywords:** Health services, Older people, Primary care

## Abstract

**Background:**

This paper presents insights into patient experiences of changes in workforce composition due to increasing deployment in general practice of practitioners from a number of different professional disciplines (skill mix). We explore these experiences via the concept of ‘patient illness work’; how a patient’s capacity for action is linked to the work arising from healthcare.

**Methods:**

We conducted four focus group interviews with Patient Participation Group members across participating English general practitioner practices. Thematic analysis and a theoretical lens of illness work were used to explore patients’ attempts to understand and navigate new structures, roles and ways to access healthcare.

**Results:**

Participants’ lack of knowledge about incoming practitioners constrained their agency in accessing primary care. They reported both increased and burdensome illness work as they were given responsibility for navigating and understanding new systems of access while simultaneously understanding new practitioner roles.

**Conclusions:**

While skill mix changes were not resisted by patients, they were keen to improve their agency in capacity to access, by being better informed about newer practitioners to accept and trust them. Some patients require support to navigate change, especially where new systems demand specific capacities such as technological skills and adaptation to unfamiliar practitioners.

## Introduction

The current workforce crisis in primary care in the UK has been described in terms of a shortfall in the number of general practitioners (GPs) to provide healthcare for a growing and ageing population. To address this shortfall, attention has been focused upon changing the occupational mix and skillsets of the primary care workforce through the employment of practitioners from a wide range of healthcare disciplines.^1^^,^^2^ A national vision for a transformed NHS based on new models of care was set out in the NHS Five Year Forward View in 2014 and refreshed in 2017^3^^,^^4^ with details set out in General Practice Forward View, published in April 2016.^5^ This proposed the creation of a minimum of 5000 new non-medical roles in primary care in the UK where ‘*wider members of the practice-based team will play an increasing role in providing day-to-day coordination and delivery of care*’ (p.7). Five roles were initially implemented: clinical pharmacists, physician associates, paramedics, physiotherapists and social prescribers. The focus on these roles was based on the availability of practitioners, the strength of practice demand for practitioners and the belief that these roles would reduce GP workload and create additional capacity. From April 2020 reimbursement increased to 100% and a further six roles were added: pharmacy technicians, care co-ordinators, health and well-being coaches, dieticians, podiatrists and occupational therapists.^6^ While patients are less familiar with these newer practitioners in general practice, increasing numbers are now being employed to address the shortage of GPs.^7^

In terms of the current socio-political landscape in England, the enhancement of healthcare that may be achievable through increased skill mix aligns with patient-centred reforms to support people to age in place, by shifting care from more distant centres to community-based settings^8^ and New Public Management policy frames patients as empowered consumers who can exercise choice. As skill mix introduces new opportunities for patients to choose between practitioners, patients become ‘responsibilized’ or forced to take a more active role in choosing the right option for their care and to discipline their behaviour to fit within these prevailing norms.^9–11^ Patient experiences are subsequently redefined by their competency to choose. However, the assumption that choice increases autonomy and empowers patients may not apply since patients may prefer health professionals to make care-related choices, especially when they are in pain or vulnerable.^12^

In this paper, we explore skill mix change from the patient’s perspective. Making arrangements to access healthcare can be considered as a form of patient ‘work’, or as tasks that are additional to their responsibilities for undertaking self-care, enduring symptoms and undergoing treatment that have previously been characterized as ‘illness work’.^13^^,^^14^ May et al.’s ^14^ framework, which considers the cognitive, material and social capacity (the resources available to the patient) and functional performativity (the degree to which patients possess the capacity to meet the demands of care), can therefore be considered relevant. Patients face increasingly complex requirements when accessing health care, as they navigate new processes and choose from a wider range of options. In addition, adapting to such systems relies on social capacity (relational networks) and functional performativity required for access. Additionally, accessing care also requires physical energy^15^ and ability to adapt to new technologies and systems that can interfere with local and contextualized knowledge.^16^ The increasing skill mix change means increasingly complex networks of relationships emerge between patients and healthcare providers, technological advancements and various structural changes,^14^ which consequently increases the work involved when patients access healthcare. For example, patients are, via skill mix changes, now provided with an array of ways that they may access an appointment, e.g. via telephone where they are triaged by a receptionist (and asked to divulge basic details of their medical complaint to the receptionist), or via practice websites or smartphone applications ‘apps’, which require patients to enter some details and an algorithm assigns the patient to an appropriate practitioner.

Research on patient experiences of skill mix changes has generally focused on patterns of care, service-utilization, patient attitudes^17–19^ or consultations with single practitioner types.^20^ Limited attention has been paid to the ‘work’ that patients are required to undertake in navigating unfamiliar roles and new systems of access. Further research is now required that looks beyond policy rhetoric of improved choice and access as merely deciding between appropriate and inappropriate help-seeking.^21^ In this context, we need to know more about the actions that patients are taking to navigate these new roles and how and in what ways their agency to achieve access is constrained or enabled.^14^ These actions, and associated understanding, link the process by which people perceive and act in their environment and, together with their interactions with others and objects (when they are expressing thoughts and acting in unknown and complex circumstances), represent both cognitive and social processes.^22–25^

This research sought to explore how changes in skill mix were experienced by patients.

## Methods

### Design

This paper is part of a wider study using a mixed-methods approach to explore the scale, scope and impact of skill mix in primary care.^47^ Workstreams included an analysis of a national dataset (NHS Digital workforce Minimum Data Set) to look at how the practitioner composition of the workforce is associated with a wide range of healthcare quality, satisfaction and cost outcomes. In addition, analysis of an online survey of practice managers at 1261 GP practices (17% of all practices in the UK) looked at motivations for employing non-GP practitioners. Researchers gathered data about how skill mix affects patients’ experiences of accessing primary care via four focus groups.

Data were analysed thematically by applying a lens of illness work^13^^,^^14^ to investigate how patients come to experience and navigate access to healthcare.^25^^,^^26^

### Data collection

Participants were recruited at four of the five study GP practices across the UK through making contact with members of their Patient Participation Groups (PPG) and providing an opportunity for other patients to join a focus group interview at their practice by responding to an invitation delivered via a patient survey (reported separately). The focus group interviews were arranged following an initial review of the survey data and provided opportunities to explore topics of interest in greater depth. We were unable to recruit patient participants at the remaining study site.

Table 1 shows the corresponding figures for completed focus groups and the time taken for each focus group:

**Table 1:** Participant characteristics and samples

Site characteristics		PPG/patient focus group (no. of participants)	Hours
A.	Early adopter of skill mix, semi-rural, with a large elderly population and large number of care homes, corporate approach to organizing care, list size ~11 000.	7	1
B.	Late adopter of skill mix, semi-rural in the centre of a small town, with large elderly population and large number of care homes, local ‘cottage’ hospital recently closed, list size ~14 000.	2	1
C.	Early adopter of skill mix, urban/suburban multi-practice sites incorporating an urgent care centre at the larger site, list size greater than 60 000.	0	0
D.	Early adopter of skill mix, remote, rural town, affluent, with a single, recently built practice serving the whole town (following historic mergers), with other health services at a distance but ‘step-down’ beds and district nurses on site, list size ~17 000.	12	1
E.	Early adopter of skill mix, suburban practice based in very small premises in a large city with easy access to other primary care services e.g. walk-in centres and hubs, list size ~10 000.	8	1
**Totals**		*N* = 29	4

### Analysis

Focus group conversations were transcribed verbatim by a University of Manchester approved transcriber. One researcher (ED) conducted an initial analysis of the focus groups thematically using familiarization with the data, free coding and identifying themes as described by Braun and Clarke ^26^. An initial coding framework was produced following discussion with project team members to organize the data into meaningful groups for each transcript. Once all individual interviews were analysed, all codes were listed and categorized into meaningful groups and then refined to form themes and subthemes.

### Burden of treatment and illness theory

Burden of Treatment Theory aims to understand how a patient’s capacity for action is linked to and interacts with the work arising from healthcare. It focuses on the work that patients and their networks do in accessing and utilizing health care and therefore can provide insight into adherence in treatment and healthcare.^14^ It refers to the burden of treatment itself, which includes but is not limited to the ongoing management of the condition itself including utilization of health care settings, the efforts required to manage this, the work the patients undertake in achieving this and their capacity to undertake this work. The theory recognizes the predominance of multi-morbidity and chronic conditions, where utilization of health care is ongoing and long-term and the growing demands and expectations on patients to therefore organize and coordinate much if not most of their care, requiring a whole host of expertise and motivations to achieve this. Patients depending upon their capacity can struggle with this and so this often results in additional treatment and illness burden or work, sometimes leading to disengagement or non-compliance with health care services if demands upon them become overwhelming.^14^

The framework examines both capacity of individuals and relational networks and how these affect interactions with healthcare. Capacity for action is impacted by relational networks, that is, enhanced relational networks increase capacity for action or here, increase capacity to engage with primary care. Relational networks refer to social skills (ability to co-ordinate/co-operate with others) and social capital (ability to access information/material resources). Relational networks between agents (individuals or groups) and contexts (diverse technical, professional and organizational structures in healthcare systems) are both structured and fluid and so this shapes opportunities to use health systems.^14^

**Fig. 1:**
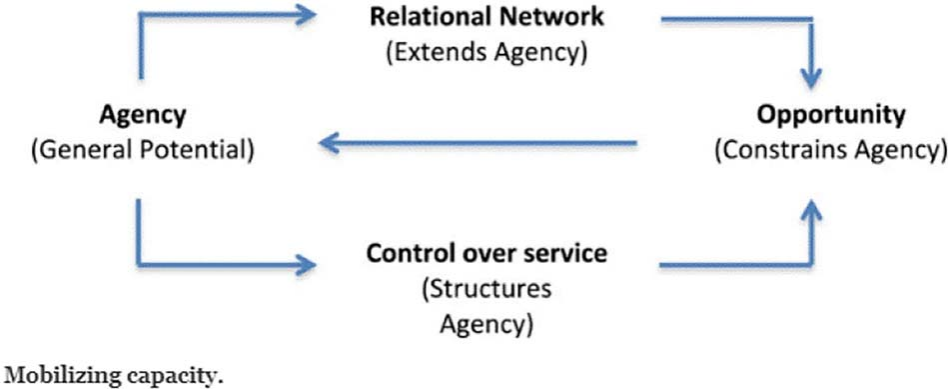
Components of capacity. Source: May et al. ^14^.

Figure 1 is taken from May et al’s ^14^ framework and shows the structure for healthcare utilization, showing how agency (the general potential of the individual patient or patient group) is mediated by their patient’s relational network and via controls (what providers do to determine content of a service). In turn, this frames what health care opportunities are available or not available to the patient, and feed back to structure the patient’s potential (agency) to utilize the service/health care. As such it is not only about an individual’s motivations or expertise, it is also about consistent and changing relational networks, contexts, information and tools that are available to patients to impact their capacity to access and engage with a service.

All data were cross-compared looking at responses from individual sites and responses as a whole. Data analysis was refined following thematic analysis in discussion with team members and utilizing an illness work framework, referring to the elements described above. Many of the themes related to ‘burden’ and illness ‘work’, both implicitly and explicitly, and so May et al’s ^14^ framework of ‘Burden of Treatment Theory’ was used to develop a deeper analysis, by categorizing different themes into meaningful groups in this framework. Two Primary Care Research in Manchester Engagement Resource (PRIMER) Patient and Public Involvement and Engagement (PPIE) members (AD and JH) contributed patients’ perspectives in all aspects of the group’s work including the development of topic guides and by assisting with interpretation in analysis e.g. thematic grouping and write up of the findings.

## Results

The illness work framework lens was helpful in understanding the ways that patients attempted to navigate unfamiliar changes (control changes in services related to skill mix which structures agency), the potential patients had to do this (agency) and the impact of opportunities (such as age) and relational networks upon agency (social skills and capital such as information on how to access the new practitioners and appointments). Patients framed the changes in relation to how they functioned as patients; by the changes that they observed within their existing structures of access, by the work required to accept different practitioners and largely by the new work required of them to understand, access and navigate care in this new context. Understanding these changes was a work in itself. Change was not framed as a facilitator of choice, but was enforced or associated with obligations. Patients were therefore responsibilized to take on additional illness work, which is presented under key emerging themes: (1) Minimal communication and increased work; (2) Navigating new systems of access; (3) Patient compromises: early appointments versus preferred practitioner and (4) Developing trust in new relationships.

1. **Minimal communication and increased work**

Focus group findings revealed participants often described how skill mix changes had not been communicated effectively to them. Where they were aware of the changes, they described what was interpreted as having to undertake further ‘work’ to make sense of the changes because information was minimal and unclear. This meant that the task of translating what skill mix changes meant, in terms of access, was implicitly transferred to patients who, in striving to understand the changes, were required to do increased work, constraining their agency to access healthcare. Patients discussed facing a ‘catch 22’ situation in obtaining access to primary care: patients needed to be regular attendees to be aware of changes and understand what the changes mean, but to gain access to care patients required a certain level of knowledge and understanding of how to navigate the changes. This highlights the complex and invisible nature of work to access healthcare being undertaken by patients:

*Because she so rarely comes to the doctors, she didn’t know the system at all.*

*ID12 -Site A, PPG Focus Group*

Participants pondered how communication of change could best be achieved, in essence, responsibilizing themselves with the task of easing this additional workload for themselves and other patients. Participants expressed differing opinions about how practices could bolster patients’ abilities to access this information, including discussion of the best form of communication to convey the information:

Education is the thing, it's really important that patients understand. And actually, you have to know how the system works...perhaps more effort could be put into patient education or providing information. But it's no good bombarding people with hundreds of leaflets. So, it's a tricky thing, you've got to find a way of impacting people when it affects them.

ID12 -Site A, PPG Focus group

There was no mention of whom patients expect the responsibility to educate patients should fall on to. At two sites (site A and D, both earlier adopters of skill mix), some participants discussed how recent improvement of the practice website meant they could now easily access information regarding the changes, improving patient’s social capital and capacity for action and therefore their functional performativity (the degree to which patients possess the capacity to meet the demands of care).

This provided increased opportunity for agency in access, by including descriptions of what the new practitioners could do. This emphasizes the relevance of changing relational networks and social capital and indicated the need for a symbiotic relationship, where patients will engage in trying to understand how best to minimize their new workload around access to health care but that they do need support from the system actors to achieve understanding of skill mix changes. Provision of ‘*readily available’* information provided opportunity for enhancing patient agency; it helped with understanding skill mix, reducing the additional illness work and improving their ability to access care with less work than had been previously required before the website was updated.

At site D, simple amendments to the telephone messaging service such as leaving detailed messages on what to do to book appointments and further training for receptionists in supporting patients improved their understanding of skill mix changes. This emphasized the mediating effect of educating patients about change by decreasing the work required of them to access care and meant they were better able to adjust to the new situation.

However, concerns were often raised regarding communication of changes with those who were not regularly accessing primary care, in particular older adults who it was felt were also less likely to use online resources. This represented how agency can be constrained and subsequently how patients may then have reduced functional performativity and agency to access care:

There’s still a lot of people even older than us who haven’t ever been on the internet, don’t know what to do with it even*.*

ID22 – Site B, PPG Focus group

In addition to limited technological abilities or material capacity that could potentially affect older adults, concern for other vulnerable groups such as those with learning disabilities was reflected across all focus groups.

2. Navigating complex new systems of access

Practices are developing an array of novel ways to enable patients to access a wider range of different practitioners, but this meant that navigation had become quite complex. Patients reported being required to choose from, for example, a telephone conversation with a receptionist (often via selecting from a number of choices via a telephone messaging service), a triage appointment booked online via the practice website or utilizing an app.

Across all of the sites, participants described accessing primary care as onerous and fraught with obstacles demanding new cognitive, social and material capacity. They had to develop new technological know-how in relation to the apps and websites and struggled to understand whether the newer practitioners could adequately assess their often multiple needs and/or comorbidities, demonstrating links between education and competence. This again highlights the increased work experienced by patients to obtain access as well as highlighting their anxieties.

Poor communication and limited patient education about these access points (where social, material and cognitive capacity converge as pertinent) were again identified as limiting factors. PPG members have attempted to resolve these new challenges to decrease this workload for others:

I rang [receptionist] up and said ‘where’s this online triage?’ ‘It’s on the homepage’. ‘No, it’s not’. ‘It’s there’. ‘Well, that’s not very big is it?’ I think the only way that we can educate people is by doing it on the website and the screens, but maybe there’s a need if money’s available to do a mail drop*.*

ID22 – Site B, PPG Focus group

Patients reported inequity within the newer arrangements in relation to the patient’s material and cognitive/technological capacity (e.g. whether they had internet access or not and/or were able to navigate the internet/practice website/app), since successfully gaining access depended on which type of access you were using. It was noted that if you were able to use the website and app, you could effectively screen both (and also still telephone to book an appointment) to find an appointment more quickly/easily and so patients able to do this were put at an advantage compared with other patients who could not. Such inequity in capacity impacted patient’s agency in accessing the service, for example the website was perceived to be ‘hard work’ and so access was ‘unfair’:

R3: If you go online and look at the appointment system it's generally, I would say from my experience, three or four weeks before you can get an appointment…I thought the idea of the apps was actually to reduce that pressure on the receptionists. Well, in that case, it's not doing that is it because if you want an appointment, you're not going to use the app. It does make it hard work… it seems a little bit unfair because people like us, if you're quite happy doing things online, you can sort of play the system, if you like, you can look on one, look on another and find an appointment. But there are a lot of people who can't do that*.*

ID46 – Site D, PPG Focus group

The previous typical approach was that a patient would be asked to provide some information to a receptionist, who would then offer an appointment with a practitioner they thought could deal with that problem, possibly supplying additional information to justify a different appointment/practitioner type. The receptionist would then respond in whatever way they felt justified by the problem and by the appointments that were available and try to find some accommodation that the patient would accept. A lack of understanding of the newer ways to access an appointment meant it was often criticized by participants as not adequately capturing patients’ medical histories and they were largely unaware as to whether their appointment was with an appropriate practitioner until after the consultation had taken place. This had at times resulted in wasted appointments, led to further work to access a subsequent appointment and reduced trust in the changes:

My husband’s got multiple health problems, and I don't know, I think every time he sees a nurse they go, ‘I think you need to see the doctor’*.*

ID55 – Site E, PPG Focus group

PPG members noted that if they found the new arrangements and roles confusing to navigate, this probably meant tasks, such as navigating the new systems and understanding the new roles, would be even more difficult for patients who were neither PPG members nor regular attendees in primary care.

It was also acknowledged that it was not ideal for patients to first receive information about these changes to access when they are unwell and arguably less able to process information. Such responsibilization of patients to navigate these changes was perceived as increasing the burden of accessing healthcare:

R9: I think what I'm aware of personally and what I'm hearing now is that people are feeling, those of us who have the wherewithal to do it, we have to take personal responsibility for our own health*.*

ID46—Site E, PPG Focus group

3. Patient compromises: early appointments versus preferred practitioner

Due to the skill mix changes, patients were required to use a new appointment booking system. Previously, this had been to call and speak to a receptionist and book an appointment with a GP. It now required either calling to speak to a receptionist and providing details about their medical complaint to the receptionist (not previously required) or navigating an appointment via the smartphone app or practice website via a set of questions that would then match them to an appropriate practitioner. Participants reported that although new access to appointments was difficult to navigate, appointments were largely perceived to be more readily available as a result of increased skill mix. Participants expressed increased acceptance of skill mix on the basis that it was better to have an appointment with someone than to be unable to see anyone. Moreover, there was an assumption that an appointment with any practitioner could lead to issues being escalated and getting ‘*an appointment quicker’* with a GP. *ID22—Site B, PPG Focus group.*

Patients were aware that skill mix changes were a consequence of broader changes within the NHS and viewed this as an inevitable change contributing to additional patient work that was appraised as a compromise that they had to ‘*accept’*. *ID46—Site D, PPG Focus group.*

In contrast to health policy messages, skill mix was not seen as an opportunity to enable better choice of practitioner and as improving access: instead, waiting times for accessing an appointment could only be reduced if patients were willing to *forgo* having a choice of practitioner, the compromise often being to accept a consultation with someone other than a GP as this patient succinctly stated:

Is it too harsh to say beggars can't be choosers?

ID46 – Site D, PPG Focus group

4. Developing trust in new relationships

Focus groups revealed that once patients had overcome the issues of access and worked to develop new relationships or changing relational networks, they were largely happy to consult with the newer practitioners. Trust was an important aspect in constructing these new relationships but, because of limited information regarding the newer practitioners, building trust was often delayed until the point of consultation.

While patients were initially unsure about the skills of different practitioners, they reported being happy once they had experienced a consultation with a new practitioner.

Patients drew on past experiences of consultations as they encountered newer types of practitioners. Focus groups reported that patients were particularly satisfied with physician associates and advanced practitioner nurses who, in comparison with GPs, provided longer consultations when they considered the patient as a ‘*whole*’ rather than dealing only with one *‘ailment’*. *ID55 -Site E, PPG Focus group.*

Participants reported positively on consulting with newer practitioners who had reached the limit of their knowledge, acknowledged this and sought help from a more senior or specialized practitioner for advice. This was viewed by the patients as a key component in improving trust and brought a ‘*sense of relief’*, improving acceptance:

I think one of the things about seeing the PA [Physician Associate] is the fact that if they're not comfortable you will be referred to a GP and I think that's really important for patients to know. They don’t just bumble along doing things by the rule but if they're not happy they’ll put their hand up and say, ‘I'm out of my depth, you need to go and see a doctor,’ and ‘you…well stay there and I’ll go and talk to them’. I've seen that happen and I think that gives you enormous sense of relief…

ID55 -Site E, PPG Focus group

However, as patients reflected upon their changing relationships with GPs, this was at times conceptualized as burdensome, as a grieving process, and fraught with concerns about the continuity of care and how this would impact upon the safety of patients:

I think there would need to be some data over a few years obviously as to the risks involved with the nurse practitioners, whether there's any, you know, records of things they've missed, which then obviously the patient has gone on and maybe had a serious illness which was missed because they saw a nurse rather than a doctor. I mean, that's really the basis of it, isn't it, as to how safe it is*.*

ID46—Site D, PPG Focus group

## Discussion

### Main finding of this study

Skill mix implementation seeks to address workload pressures in primary care. Our analysis shows that the picture is complex and that skill mix changes may unintentionally contribute to increased patient work and if changes are not communicated effectively, this may constrain patient agency in accessing an appointment.^14^

### What is already known on this topic and what this study adds

Rather than it being simply that the extended size of skill mix teams makes choice of practitioner more difficult for patients^27^ our findings are consistent with previous evidence that patients have little knowledge about the roles of different practitioners in primary care.^28^ Lack of information about skill mix changes, coupled with advances in technology and limited patient ability, literacy and skills to use apps and websites and lack of education and capacity building around these technologies, meant that patients had increased work to access care.^14^ Like Dent and colleagues^9^^,^^10^ we found evidence that the absence of adequate information placed additional responsibility to negotiate complex new access arrangements and new relationships onto patients rather than providing the benefits of increased choice. Patients were tasked with modifying their behaviour to fit within new prevailing norms within primary care^11^ but patient empowerment in the manner intended by that policy was not evident.^29^ Instead, responsibilization in the absence of adequate information added to the work required of patients seeking healthcare and exacerbated the existing burden of treatment and inequalities for patients who may not have the capacity (cognitive, social and material) to navigate changes.^14^

Participants raised concerns about adverse consequences for groups such as those previously identified as less able to navigate system changes, for example those with complex needs.^30^^,^^31^ We note that while Corscadden et al^32^ found older patients (aged 65+) were somewhat protected from experiencing difficulties with multiple barriers before reaching primary care, our findings are better aligned with reports that technological advances may impact negatively on patients’ capacity to use new systems.^15^^,^^33–35^ In addition to older patients and vulnerable groups, unwell patients were perceived as not able to navigate changes.^12^

Patients framed acceptance of the skill mix changes as a compromise they made to gain access and at times even as an obligation (to accept). This added to the challenges of self-care-oriented policies, meaning that all patients need to be highly skilled to negotiate many social and now technological processes to access primary care.^14^^,^^36^ This underlines the need to consider the symbolic properties of such new technologies, with their connotations of ‘youth and progress’,^16^^, p.^^7^ and a need for more in-depth consideration of how use of such technologies, such as by older adults, who may not have the necessary abilities to use the technologies, may create additional burden.

Participants felt that particularly for older adults, the work required to build trust and construct new relationships while experiencing the loss of existing relationships in primary care was challenging.^37^ While patients valued continuity of care, they appeared largely happy with their new relationships once they had experienced consultations with new practitioners, particularly with PAs and nurses. Previous studies suggest that continuing experience of new practitioners should improve satisfaction and acceptance of skill mix.^20^^,^^38^ Improvement in the availability (as opposed to access to) appointments was welcomed and is consistent with previous studies.^39–45^

The findings overall have highlighted the impact of uncertainty as a result of changes in operational and structural elements of healthcare that may have unintended consequences for the way that patients access (or fail to access) health care, which requires further exploration to understand healthcare utilisation.^14^^,^^46^

Restrictions introduced during the Covid-19 pandemic have increased the role of digital technologies in delivery of health care. Future research should explore the adaptation of these technologies to meet the complex requirements of primary care providers and patients (rather than the other way around), to minimize additional burden and inequity for vulnerable groups.

### Limitations of this study

Focus groups from four GP practices in the UK were included with more participants in the focus groups from early adopters of skill mix and due to this and the qualitative nature of this component of the study, these results are not generalizable and are therefore limited in this respect.

## Conclusion

Changes in the primary care workforce and processes can have a significant and inequitable impact on patients. To minimize the additional work required of patients and improve trust in newer practitioners, clear communication of information is needed, using appropriate forms and with adequate support to help patients understand new systems and what newer types of practitioners can do. Longer term consequences should be considered due to potential impacts on continuity of care, safety and equitable access.

## Data Availability

The data obtained from participants in this study is not available due to the restricted scope of consent obtained from participants and related ethical restrictions.

## Notes

**Elizabeth Dalgarno,** Lecturer in Health Sciences

**Imelda McDermott,** Research Fellow

**Mhorag Goff,** Research Associate

**Sharon Spooner,** Clinical Lecturer

**Anne McBride,** Professor of Employment Studies

**Damian Hodgson,** Professor of Organisational Studies

**Ailsa Donnelly,** PPG member

**Judith Hogg,** PPG member

**Kath Checkland,** Professor of Health Policy and Primary Care
